# Intricate MIB1-NOTCH-GATA6 Interactions in Cardiac Valvular and Septal Development

**DOI:** 10.3390/jcdd11070223

**Published:** 2024-07-15

**Authors:** Rebeca Piñeiro-Sabarís, Donal MacGrogan, José Luis de la Pompa

**Affiliations:** 1 Intercellular Signaling in Cardiovascular Development & Disease Laboratory, Centro Nacional de Investigaciones Cardiovasculares (CNIC), Melchor Fernández Almagro 3, 28029 Madrid, Spain; rebeca.pineiro@cnic.es; 2 Ciber de Enfermedades Cardiovasculares, Instituto de Salud Carlos III, Melchor Fernández Almagro 3, 28029 Madrid, Spain

**Keywords:** CHD, BAV, VSD, genetic interactions, GATA6, MIB1, NOTCH1

## Abstract

Genome-wide association studies and experimental mouse models implicate the *MIB1* and *GATA6* genes in congenital heart disease (CHD). Their close physical proximity and conserved synteny suggest that these two genes might be involved in analogous cardiac developmental processes. Heterozygous *Gata6* loss-of-function mutations alone or humanized *Mib1* mutations in a NOTCH1-sensitized genetic background cause bicuspid aortic valve (BAV) and a membranous ventricular septal defect (VSD), consistent with MIB1 and NOTCH1 functioning in the same pathway. To determine if MIB1-NOTCH and GATA6 interact in valvular and septal development, we generated compound heterozygote mice carrying different Mib1 *missense* (*Mib1^K735R^* and *Mib1^V943F^*) or *nonsense* (*Mib1^R530X^*) mutations with the *Gata6^STOP/+^* heterozygous null mutation. Combining *Mib1^R530X/+^* or *Mib1^K735R/+^* with *Gata6^STOP/+^* does not affect *Gata6^STOP/+^* single mutant phenotypes. In contrast, combining *Mib1^V943F/+^* with *Gata6^STOP/+^* decreases the incidence of BAV and VSD by 50%, suggesting a suppressive effect of *Mib1^V943F/+^* on *Gata6^STOP/+^*. Transcriptomic and functional analyses revealed that while the EMT pathway term is depleted in the *Gata6^STOP/+^* mutant, introducing the *Mib1^V943F^* variant robustly enriches this term, consistent with the *Mib1^V943F/+^* phenotypic suppression of *Gata6^STOP/+^*. Interestingly, combined *Notch1* and *Gata6* insufficiency led to a nearly fully penetrant VSD but did not affect the BAV phenotype, underscoring the complex functional relationship between MIB1, NOTCH, and GATA6 in valvular and septal development.

## 1. Introduction

Bicuspid aortic valve (BAV) is the most prevalent congenital heart defect, affecting up to 2% of the population, and is characterized by two asymmetrical leaflets instead of the three leaflets found in normal aortic valves [1,2]. BAV individuals often do not show symptoms and are usually diagnosed incidentally unless BAV is linked to significant aortic valve dysfunction, including stenosis and regurgitation [3,4,5,6]. Importantly, BAV disease in younger patients is associated with aortic medial defects that result in a higher prevalence and accelerated rate of aortic dilatation, increasing the risk of lethal dissection or rupture [7,8]. Aortic valve development entails the formation of endocardial cushions, their cellularization through epithelial-to-mesenchymal transition (EMT), and remodeling by mesenchymal compaction. Concomitant alignment and fusion of the endocardial cushion mesenchyme is required to ensure cardiac septation [9,10].

Leaflet orientation in BAV varies relative to the coronary arteries, resulting in different classifications, which depend on the number of raphe [11,12]. In rodents, two main types of BAV have been described, involving the right and left leaflets (R-L) and, more commonly, the right and non-coronary leaflets (R-N) [13]. Mechanistically, RL-BAV may be caused by defective cardiac neural crest cells (CNCCs) patterning of the outflow tract (OFT) [13]. RN-type BAV may result from defective endocardial cushion formation, presumably because of impaired EMT [14].

BAV is inherited in a dominant manner, with incomplete penetrance and variable expressivity [2,15]. Genome-wide association studies have identified chromosomal regions 9q, containing *NOTCH1*, and 18q, harboring *GATA6* and *MIB1*, as linked to BAV [16]. Mouse *Gata6* and *Mib1* genes are closely associated within 300 kb of DNA in a region of conserved synteny with the human locus 18q11.2 region, which is frequently implicated in cardiac abnormalities.

GATA6 is a key transcription factor that plays a crucial role in various developmental processes, including initiating the EMT during mesoderm development and promoting the migration of CNCCs [17,18]. *GATA6* mutations have been reported in a wide spectrum of CHD, including BAV [19,20,21]. *Gata6* haploinsufficiency causes BAV [20,22], whereas deletion in CNCCs causes aortic arch defects, suggesting a requirement in vascular smooth muscle cell differentiation [23,24].

MIB1 is an E3 ubiquitin ligase required for activation of the NOTCH pathway [25]. *NOTCH1* and *MIB1* mutations have been reported in BAV [26,27,28]. *Mib1* inactivation in mice broadly recapitulates NOTCH loss of function cardiac phenotypes, including BAV [14,29,30]. Humanized mouse models for the *Mib1^R530X^ nonsense* or *Mib1^V943F^ missense* mutation [28,31] or the *Mib1^K735R^ missense* mutation [28] develop BAV in a Notch-sensitized background [28,32], indicating that valve morphogenesis is highly MIB1-NOTCH dosage-sensitive.

*GATA6* and *MIB1’s* close physical proximity on chromosome band 18q11 suggests their involvement in shared developmental mechanisms. In this study, we investigated the genetic interactions between *Gata6* and *Mib1* and found a complex functional relationship between GATA6 and MIB1-NOTCH, which provides valuable insights into the genetic determinants of BAV, VSD, and related cardiac anomalies.

## 2. Materials and Methods

### 2.1. Ethics Statement

Animal studies were approved by the CNIC Animal Experimentation Ethics Committee and by the Community of Madrid (Ref. PROEX 155.7/20). All animal procedures conformed to EU Directive 2010/63EU and Recommendation 2007/526/EC regarding the protection of animals used for experimental and other scientific purposes, enforced in Spanish law under Real Decreto 1201/2005.

### 2.2. Mouse Strains

Mouse strains used in this study are as follows: *Gata6^STOP/+^* [22], *Mib1^R530X/+^* [33], *Mib1^V943F/+^* [34], *Mib1^K735R/+^* [35], *Notch1^KO/+^* [36]. Genotyping primers are shown in Table S1.

### 2.3. Tissue Processing for Histological Procedures

For histological procedures, whole embryos or torsos were fixed in 4% paraformaldehyde (PFA, Electron Microscopy Sciences, 50980487, Hatfield, PA, USA) overnight at 4 °C. Paraffin-embedded embryos/torsos were cut into 7 μm sections. Hematoxylin and eosin (H&E) staining was performed according to standard protocols.

### 2.4. Microscopy and Confocal Imaging

Brightfield imaging was performed using an Olympus BX51 Microscope (Tokyo, Japan) and Olympus cellSense software (Version number 1.7). Images were processed in Adobe Photoshop Creative Suite 5.1.

### 2.5. Statistical Analysis

Sample sizes, statistical tests, and *p*-values are specified in the corresponding figure legends and corresponding subsections of Section 2. For comparisons between two groups, a mean ± SD is represented, and an unpaired two-tailed Student’s *t*-test was performed. For experiments comparing two groups of categorical variables, a mean ± SD per group is represented, and a Chi-Square test was performed. Differences were considered statistically significant at a *p*-value < 0.05 (Table S2). Statistical analysis and graphical representation were performed using GraphPad Prism, version 8.

### 2.6. RNA-Seq

RNA was isolated from E11.5 *Mib1^V943F/+^ Gata6^+/STOP^* and control OFT samples. Samples were distributed in three pools of four pairs of OFT per genotype. Tissue was homogenized with a pestle mechanical homogenizer, and RNA was extracted with an Arcturus PicoPure RNA Isolation kit (Thermo Fisher Scientific, Norristown, PA, USA, KIT0214). RNA libraries were prepared using the NEB Next Ultra II Directional RNA Library Prep Kit (Ipswich, MA, USA) and sequenced in a Nextseq 2000 Illumina sequencer (San Diego, CA, USA) using a 60 bp single-end elongation protocol. Sequenced reads were QC and pre-processed using cutadapt v1.18 [37] to remove adapter contaminants. The resulting reads were aligned, and gene expression was quantified using RSEM v1.2.3 [38] over mouse reference GRCm38 with Ensembl genebuild. Differential gene expression was analyzed with the EdgeR R package (v3.32.1 on R 4.0.3) [39]. Genes with 1 count per million (cpm) in at least 3 samples were defined as expressed and retained for later analysis. Counts were normalized by the TMM method. Differential gene expression was tested using a generalized linear model as implemented in the EdgeR package. Genes showing altered expression with an adj. *p*-value < 0.05 were considered differentially expressed. The set of differentially expressed genes was used for functional analysis with Ingenuity Pathway Analysis Software (version 111725566, Qiagen-IPA, Redwood City, CA, USA) [40], where we used a Benjamani–Hochberg adjusted *p*-value < 0.05 for significance. Gene Set Enrichment Analysis (GSEA) was performed with GSEA on the complete set of expressed genes against the Hallmark term database. A NOM *p*-value < 0.1 was used to select for significantly enriched gene sets.

## 3. Results

### 3.1. Allele-Specific Mib1 Interaction with Gata6^STOP/+^

**Figure 1:**
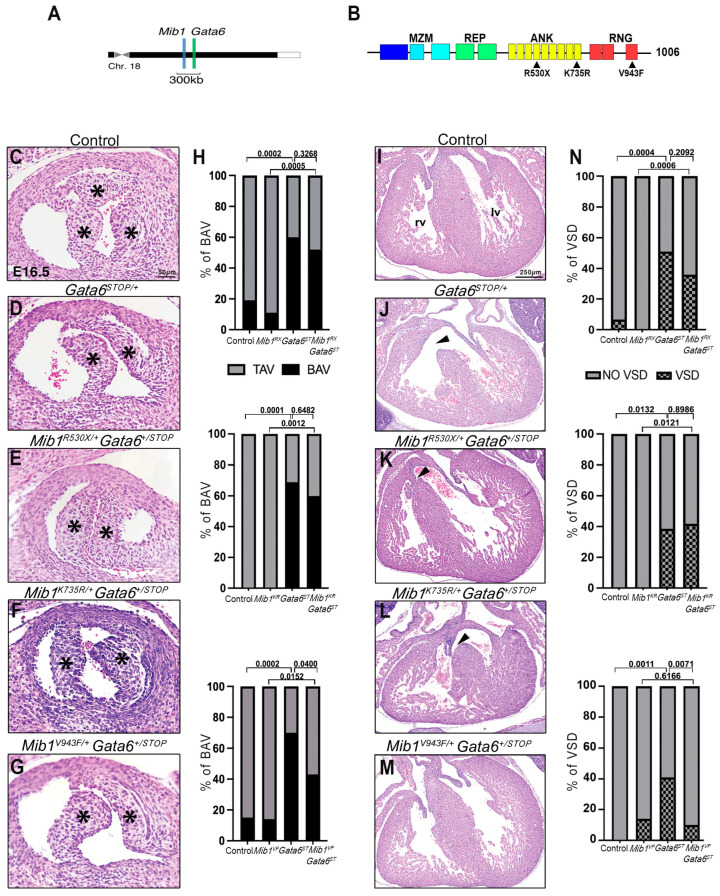
Allele-specific *Mib1* interaction with *Gata6^STOP/+^*. (**A**) Schematic representation of the chromosome 18 region harboring *Mib1* and *Gata6* genes. (**B**) Graphical representation of the MIB1 domain organization and location of the identified MIB1 variants. MZM: Mib-Herc2 domain 1 + ZZ finger domain + Mib-Herc2 domain 2. REP: Mib Repeats 1 and 2. ANK: Ankyrin repeats 1−9. RNG: Ring domains 1−3. (**C**–**G**) H&E staining on aortic valve sections in control (**C**), *Gata6^STOP/+^* (**D**), *Mib1^R530X/+^ Gata6^+/STOP^* (**E**), *Mib1^K735R/+^ Gata6^+/STOP^* (**F**), and *Mib1^V943F/+^ Gata6^+/STOP^* mice (**G**) at E16.5. The asterisk (*) indicates the position of the leaflets. (**H**) Quantification of BAV percentage. Statistical significance was determined by the Chi-Square test. (**I**–**M**) H&E staining on ventricular chamber sections in control (**I**), *Gata6^STOP/+^* (**J**), *Mib1^R530X/+^ Gata6^+/STOP^* (**K**), *Mib1^K735R/+^ Gata6^+/STOP^*(**L**), and *Mib1^V943F/+^ Gata6^+/STOP^* mice (**M**) at E16.5. Arrowheads indicate VSD. rv, right ventricle. lv, left ventricle. (**N**) Quantification of VSD percentage. Statistical significance was determined by the Chi-Square test.

*Mib1* and *Gata6* are in close proximity in the chromosome 18q11.2 region (Figure 1A). To determine if *Mib1* and *Gata6* interact genetically in valvular and septal development, we generated compound heterozygote mouse lines by combining in trans three different humanized *Mib1* inactivating alleles (*Mib1^R530X^, Mib1^K735R^, Mib1^V943F^*) [28,31,32] with a *Gata6*-null allele (harboring the Gata6^V291X^ mutation, from now onwards *Gata6^STOP^*) [22].

*Mib1^R530X^* is a *nonsense* mutation that results in premature termination, nonsense-mediated decay [31,41], and haploinsufficiency in humans [31] (Figure 1B). At E16.5, *Mib1^R530X/^*^+^ *Gata6^+/STOP^* double heterozygous mice, in which the *Mib1^R530X^* and *Gata6^STOP^* mutations are in trans configuration, developed an RN-BAV with 50% penetrance (14 of 28) compared to 62% penetrance (23 of 37) for the single *Gata6^STOP/+^* mutant (χ2 = 0.9615, *p* = 0.3268), and 7% (2 of 27) for the *Mib1^R530X/+^* mutant (χ2 = 12.09, *p* = 0.0005) (Figure 1C–E,H). *Mib1^R530X/+^ Gata6^+/STOP^* mutants also had VSD with 36% penetrance (10 of 28), compared to 51% VSD penetrance (19 of 37) for the single *Gata6^STOP/+^* mutant (χ2 = 1.577, *p* = 0.2092) (Figure 1I–K,N). These data indicate that *Gata6^STOP^* and *Mib1^R530X^* alleles do not interact genetically during valvular and septal development.

*Mib1^K735R^* is a *missense* mutation affecting the ankyrin repeats of the MIB1 ANK region (Figure 1B), which mediates MIB1 dimerization, suggesting that it acts as a dominant-negative mutation [28]. At E16.5, *Mib1^K735R/+^ Gata6^+/STOP^* double heterozygous mice, in which the *Mib1^K735R^* and *Gata6^STOP^* mutations are in trans configuration, developed an RN-BAV with 60% penetrance (6 of 10) compared to 69% penetrance (11 of 16) for the single *Gata6^STOP/+^* mutant (χ2 = 0.2082, *p* = 0.6482), and 0% (0 of 13) for the *Mib1^K735R/+^* mutant (Figure 1D,F,H). *Mib1^K735R/+^ Gata6^+/STOP^* mutants also had VSD with 40% penetrance (4 of 10), compared to 38% VSD penetrance (6 of 16) for the single *Gata6^STOP/+^* mutant (χ2 = 0.01625, *p* = 0.8986), and 0% (0 of 13) for the *Mib1^K735R/+^* mutant (Figure 1J,L,N). Therefore, *Mib1^K735R/+^ Gata6^+/STOP^* alleles do not interact genetically in valvular and septal development.

*Mib1^V943F^* is a *missense* mutation affecting the third RING domain of the RNG region that catalyzes ubiquitin transfer to substrate proteins [42] (Figure 1B). Previously, we demonstrated that MIB1^V943F^ has a dominant negative effect on normal MIB1 function [31]. At E16.5, *Mib1^V943F/+^ Gata6^+/STOP^* mice displayed an RN-BAV with 43% penetrance (13 of 30) compared to 70% penetrance (19 of 27) in *Gata6^STOP/+^* mice (χ2 = 4.219, *p* = 0.0400) (Figure 1G,H). *Mib1^V943F/+^ Gata6^+/STOP^* mice also developed VSD with 10% penetrance (3 of 30) compared to 41% penetrance (11 of 27) in *Gata6^STOP/+^* mice (χ2 = 7.248, *p* = 0.0071) (Figure 1M,N). Therefore, *Mib1^V943F^* has a suppressive effect on the *Gata6^STOP^* valvular and septal phenotypes.

### 3.2. Mib1^V943F^ Partially Restores EMT in Gata6^STOP/+^ Mice

**Figure 2:**
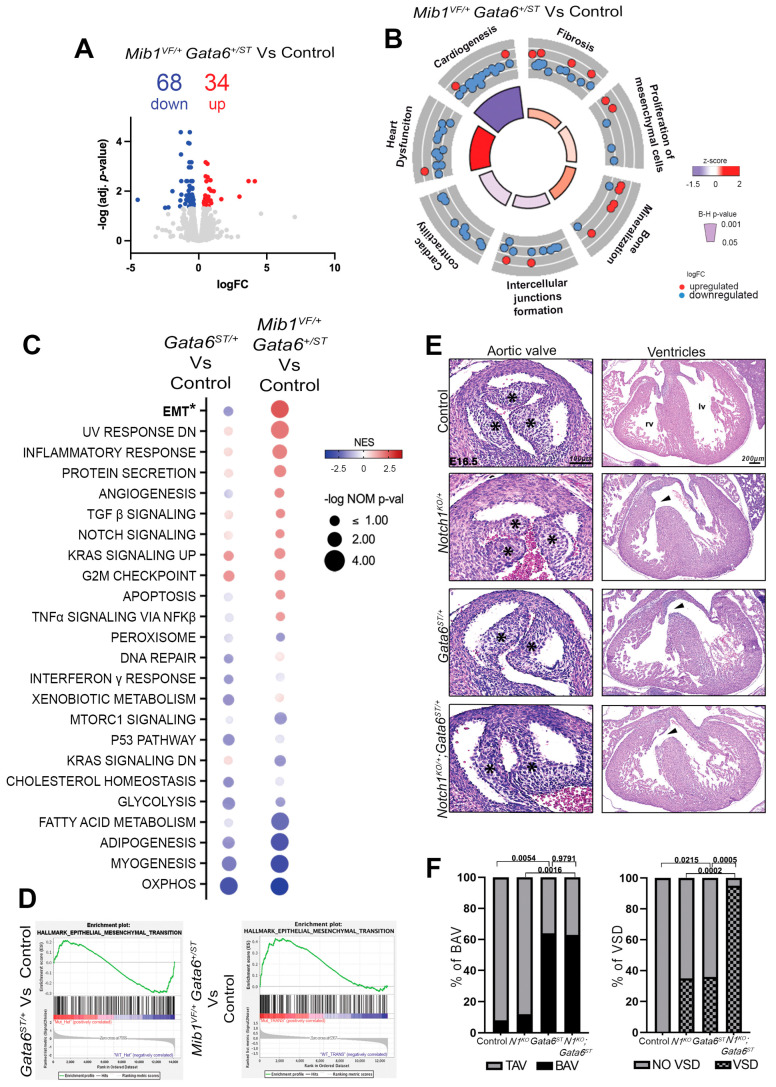
*Mib1^V943F^* partially restores EMT in *Gata6^STOP/+^* mice. (**A**) Volcano plot of the transcripts detected by RNA-seq of *Mib1^V943F/+^ Gata6^+/STOP^* vs. control contrast. Significantly downregulated and upregulated genes (adjusted *p*-value < 0.05) are labeled in blue and red, respectively. Non-differentially expressed genes are labeled in gray. (**B**) Circular plot highlighting selected Ingenuity Pathway Analysis diseases and function terms enriched in the *Mib1^V943F/+^ Gata6^+/STOP^* genotype. Red dots, upregulated genes in the pathway. Blue dots, downregulated genes in the pathway. The height of the inner circle section represents the Benjamani–Hochberg (B-H) *p*-value < 0.05 (higher is more significant), and enrichment z-score values are color-coded from positive (red) to negative (blue). (**C**) Bubble plots show 24 enriched statistically significant Hallmark gene sets in *Gata6^STOP/+^* vs. control and *Mib1^V943F/+^ Gata6^+/STOP^* vs. control contrasts by Gene Set Enrichment Analysis (GSEA). Negative logarithm of NOM *p*-value < 0.1 is represented by the size of the bubble (bigger is more significant). Normalized enrichment score (NES) is color-coded from positive (red) to negative (blue). Low-color density bubbles represent the Hallmark gene set categories that are not statistically significant in this specific comparison. (**D**) Gene enrichment profiles for the “EMT” gene set in the comparison of *Gata6^STOP/+^* (NOM *p*-value = 0.0590; NES = −1.28) and *Mib1^V943F/+^ Gata6^+/STOP^* (NOM *p*-value = 0.0001; NES = 2.02). The expression intensity is plotted on a scale from red (upregulated) to blue (downregulated). (**E**) H&E staining on aortic valve and ventricular chamber sections in controls *Notch1^KO/+^*, *Gata6^STOP/+^*, and *Gata6^STOP/+^; Notch1^KO/+^* mice at E16.5. The asterisk (*) indicates the position of the leaflets. Arrowheads indicate VSD. rv, right ventricle. lv, left ventricle. (**F**) Quantification of BAV and VSD percentages, respectively. Statistical significance was determined by the Chi-Square test.

To gain insight, we performed RNA-seq on E11.5 *Mib1^V943F/+^ Gata6^+/STOP^* mutant and control outflow tract (OFT) tissue. Differential expression analysis identified 102 differentially expressed genes (DEGs), 34 of which were upregulated and 68 downregulated (Figure 2A; Table S3). We used Ingenuity Pathway Analysis (IPA) to provide insights into the regulatory networks participating in aortic valve development (Ref. [37]). This analysis uncovered multiple enriched categories in *Mib1^V943F/+^ Gata6^+/STOP^* mice, consistent with processes expected to be altered in BAV and VSD, including “*Heart dysfunction*”, “*Bone Mineralization*”, “*Fibrosis*”, and “*Proliferation of mesenchymal cells*”. In contrast, functional categories like “*Cardiogenesis*”, “*Intercellular junctions formation*”, and “*Cardiac contractility*” were all depleted (Figure 2B).

We performed a comparative Gene Set Enrichment Analysis (GSEA) against “HALLMARK” gene sets, which are collections of predefined genes representing fundamental, well-defined biological processes [43]. Gene sets differentially enriched between *Mib1^V943F/+^ Gata6^+/STOP^* and *Gata6^STOP/+^* mutants and respective controls [22] were broadly similar, although several changes were exacerbated in compound heterozygotes (Figure 2C). For example, “UV RESPONSE DOWN”, “INFLAMMATORY RESPONSE,” and “PROTEIN SECRETION” were more significantly enriched, and “MYOGENESIS”, “ADIPOGENESIS”, and “FATTY ACID METABOLISM” were more significantly depleted (Figure 2C). However, enriched pathways evoking a proliferative defect like “G2M CHECKPOINT” and “KRAS SIGNALING UP”, and depleted metabolic pathways like “OXIDATIVE PHOSPHORYLATION” and “GLYCOLYSIS” did not deviate significantly. Importantly, “EPITHELIAL MESENCHYMAL TRANSITION” (“EMT”), the key process by which the endocardial cushions become cellularized by endocardial and neural crest-derived mesenchyme, was depleted in the *Gata6^STOP/+^* mutant but enriched in the *Mib1^V943F/+^ Gata6^+/STOP^* mutant (Figure 2C,D). These data indicate that the *Mib1^V943F/+^* mutation partially suppresses the *Gata6* haploinsufficient phenotype by enhancing EMT.

### 3.3. Compound Notch1 and Gata6 Haploinsufficiency Leads to Highly Penetrant VSD

The *Mib1^V943F/+^* suppressive effect on the *Gata6^STOP/+^* phenotype and the absence of interaction between *Mib1^R530X/+^* or *Mib1^K735R/+^* and *Gata6^STOP/+^* may depend on the dosage of NOTCH. To clarify this, we generated *Notch1^KO/+^; Gata6^STOP/+^* compound heterozygote mice (Figure 2E). Combining *Gata6^STOP/+^* and *Notch1^KO/+^* mutant alleles resulted in BAV with 63% penetrance (12 of 19), comparable to single *Gata6^STOP/+^* mutant mice (64% penetrance or 7 of 11) (χ2 = 0.0007, *p* = 0.9791) (Figure 2E,F). However, *Notch1^KO/+^*; *Gata6^STOP/+^* mice displayed VSD with 95% penetrance (18 of 19) compared with 35% penetrance (6 of 17) in *Notch1^KO/+^* mutants (χ2 = 14.27, *p* = 0.0002), and with 36% penetrance (4 of 11) in *Gata6^STOP/+^* mutants (χ2 = 12.14, *p* = 0.0005) (Figure 2E,F). Therefore, the combined haploinsufficiency of Notch1 and Gata6 leads to a high percentage of VSD but does not alter the frequency of BAV. This indicates that VSDs are particularly sensitive to the combined insufficiencies of the *Gata6* and *Notch1* genes, resulting in a more severe phenotype.

## 4. Discussion

Genetic interactions analysis aims to determine if two different genes participate in the same developmental process by combining mutations in these genes and examine if the resulting phenotype diverges from that caused by individual mutations [44]. In this study, we examined whether the combination of *Mib1-Notch1* and *Gata6* mutations had a more profound impact on valvular and septal development than the loss of Gata6 alone. Previously, we observed that *Mib1* mutations affect valvular and septal development only in a sensitized, Notch-deprived genetic background [28,32]. We identified three types of interactions between *Mib1-Notch1* and *Gata6* mutations: 1. Neutral interaction: *Mib1^K735R/+^ Gata6^+/STOP^* and *Mib1^R530X/+^ Gata6^+/STOP^* mutants show BAV and VSD frequencies similar to those of *GATA6^STOP/+^* heterozygotes; 2. Synergistic interaction: *Notch1^KO/+^; Gata6^STOP/+^* mutants exhibit a higher than expected VSD frequency compared to *Gata6^STOP/+^* heterozygotes alone. 3. Partially suppressive interaction: *Mib1^V943F/+^ Gata6^+/STOP^* mutations show lower BAV and VSD frequencies than *Gata6^STOP/+^* heterozygotes. These findings suggest that the interactions between MIB1-NOTCH1 and GATA6 are complex and may result in different biological outcomes, such as endocardial cushion formation and epithelial–mesenchymal transition (EMT), depending on the developmental context and/or temporal-spatial expression.

Our data may reflect differences due to the nature of the mutations analyzed and the NOTCH dosage, which is less stringent in the context of valvular and septal development in mice compared to humans. Specifically, the *Mib1^R530X^* and *Mib1^K735R^* mutations are predicted to be loss-of-function (null) and dominant-negative, respectively. These mutations contribute to the manifestation of valvular and septal phenotypes when combined with a *Notch1* mutation, indicating that cardiac phenotypes are sensitive to Mib1-Notch1 dosage [28,32]. This interpretation is supported by the nearly fully penetrant VSD phenotype (95%) observed in compound *Notch1^KO/+^; Gata6^STOP/+^* heterozygotes, compared to the 40% VSD frequency in *Notch1^KO/+^* mutants.

Our finding that heteroallelic *Notch1^KO/+^; Gata6^STOP/+^* haploinsufficiency does not exacerbate the BAV phenotype underscores the more significant role of GATA6 in the dosage-dependent control of aortic valve development. This is further supported by the high mutational burden of *GATA6* in human CHD [45]. Moreover, GATA6 is a key driver in the early differentiation of the second heart field [46] and the recruitment of CNCC lineages [24]. These processes collectively shape the cardiac outflow tract (OFT), potentially acting upstream of NOTCH inhibitory functions during early cardiogenesis.

Our finding that *Mib1^V943F/+^* partially restores the wild-type valvular and septal phenotype when combined with *Gata6^STOP^* is intriguing, especially considering the expected negative effects from NOTCH loss-of-function interactions. Although *Mib1^V943F^* has previously been characterized as a dominant negative mutation [31], its phenotypic expression still requires *Notch1* sensitization [28]. The underlying mechanism of this suppression involves the restoration of EMT in *Mib1^V943F/+^ Gata6^+/STOP/+^* mice, which aligns with the known roles of GATA6 and NOTCH in promoting this process.

The mechanism underlying the compensatory effect on the valvular and septal phenotypes of the *Mib1^V943F/+^* mutation when combined with *Gata6^STOP^* is unclear. Quantitative PCR experiments indicate that *Gata6* mRNA expression in the *Mib1^V943F/+^ Gata6^+/STOP^* mutants is the predicted 50%, suggesting a post-transcriptional mechanism of compensation. GATA6 interacts with GATA4 [47,48,49], GATA factors, and other cardiac factors, ie. TBX5 and/or NKX2.5 [50]. Compensation mechanisms could be explored by quantifying the protein expression levels of GATA6 and interacting partners in the compound heterozygotes.

The limitations of our study are that *Gata6* haploinsufficiency has a disproportionately large effect size on the BAV phenotype alone and that heterozygous *Mib1* mutations fail to produce phenotypes in mice unless they are sensitized by a second NOTCH mutation. Additional insight might be obtained from conditional null disruption experiments affecting the tissue-specific functions of GATA6 and NOTCH.

## Data Availability

Data are deposited in the NCBI GEO database under accession number GSE264238. The following secure token has been created to allow reviewer access while it remains in private status: wnahkyewzluprmd.

## Supplementary Materials

The following supporting information can be downloaded at https://www.mdpi.com/article/10.3390/jcdd11070223/s1.
